# Evidence‐based policy choices for efficient and equitable cervical cancer screening programs in low‐resource settings

**DOI:** 10.1002/cam4.1123

**Published:** 2017-07-14

**Authors:** Nicole G. Campos, Vivien Tsu, Jose Jeronimo, Mercy Mvundura, Jane J. Kim

**Affiliations:** ^1^ Center for Health Decision Science Harvard T.H. Chan School of Public Health 718 Huntington Avenue Boston Massachusetts; ^2^ PATH Reproductive Health Program Seattle Washington; ^3^ PATH Devices and Tools Program Seattle Washington

**Keywords:** cancer screening, cervical cancer, cost‐effectiveness analysis, health policy, HPV DNA test

## Abstract

Women in developing countries disproportionately bear the burden of cervical cancer. The availability of prophylactic vaccines against human papillomavirus (HPV) types 16 and 18, which cause approximately 70% of cervical cancers, provides reason for optimism as roll‐out begins with support from Gavi, the Vaccine Alliance. However, for the hundreds of millions of women beyond the target age for HPV vaccination, cervical cancer screening to detect and treat precancerous lesions remains the only form of prevention. Here we describe the challenges that confront screening programs in low‐resource settings, including (1) optimizing screening test effectiveness; (2) achieving high screening coverage of the target population; and (3) managing screen‐positive women. For each of these challenges, we summarize the tradeoffs between resource utilization and programmatic attributes. We then highlight opportunities for efficient and equitable programming, with supporting evidence from recent mathematical modeling analyses informed by data from the PATH demonstration projects in India, Nicaragua, and Uganda.

## Introduction

Cervical cancer is eminently preventable. The natural history of disease is well understood — that cervical cancer is caused by persistent infection with oncogenic human papillomavirus (HPV), acquired through sexual activity 1; that most HPV infections clear spontaneously within one to two years; and that a small number of infections progress to precancerous lesions which, if untreated, may become invasive 2, 3. Technologies to prevent cervical cancer are available, including (1) two prophylactic vaccines with high efficacy against HPV types 16 and 18, which cause 70% of cervical cancer cases globally 4, and a nonavalent vaccine against five additional oncogenic HPV types that together with HPV‐16,18 cause up to 90% of cases; (2) sensitive screening tests that detect oncogenic HPV infections and precancerous lesions; and (3) effective treatments for precancer 5, 6.

Yet cervical cancer is a leading cause of cancer mortality in poor women. Due to limited infrastructure and health budgets, the technological advances that can prevent, detect, and treat oncogenic HPV infections are not generally available in low‐ and middle‐income countries (LMICs), where 86% of cervical cancers occur 7, 8. Between 2006 and 2014, only an estimated 3% of females aged 10–20 years in less developed regions had received even one dose of HPV vaccine 8. While Gavi, the Vaccine Alliance, is supporting demonstration projects that may pave the way for national programs in 28 low‐ and lower‐middle‐income countries eligible for Gavi assistance 9, only seven Gavi‐eligible countries to date have begun roll‐out or are approved to receive HPV national vaccine introduction support 10. For the hundreds of millions of women in LMICs who are beyond the target age for vaccination 11, screening remains the only form of prevention.

While cytology (i.e., Pap) testing is the most common cervical cancer screening method worldwide 12 and is credited with reducing cervical cancer risk in developed countries 13, the need for frequent screening, trained personnel, laboratory infrastructure, and diagnostic follow‐up at higher level facilities leads to poor screening outcomes in low‐resource settings. An alternative screening method, visual inspection with acetic acid (VIA), is a low‐cost test that requires few supplies and no laboratory infrastructure, but has low sensitivity to detect precancer. Despite the demonstrated effectiveness of HPV‐based screening, there are no nationally scaled HPV‐based screening programs, and screening coverage with Pap testing or VIA remains low 14, 15.

To inform evidence‐based decision making and intensify a global policy focus on cervical cancer screening, we describe the challenges that confront screening programs in low‐resource settings, including (1) optimizing screening test effectiveness; (2) achieving high screening coverage of the target population; and (3) managing screen‐positive women. For each of these challenges, we summarize the tradeoffs between resource utilization and programmatic attributes. We then highlight opportunities for efficient and equitable prevention programming, with supporting evidence from recent mathematical modeling analyses informed by data from the PATH demonstration projects in India, Nicaragua, and Uganda 16, 17, 18.

## Optimizing Screening Effectiveness Given Financial and Logistical Constraints

Optimizing screening effectiveness involves selecting the screening test, delivery mechanism, and screening age(s) and interval that are associated with the greatest reductions in cancer burden while taking into account the financial and logistical constraints in a given setting. The World Health Organization (WHO) recommends, where resources are available, a “screen‐and‐treat” strategy with HPV testing and timely cryotherapy for eligible HPV‐positive women aged 30 to 49 years 19. Where resources are insufficient for HPV testing, the WHO recommends screening with VIA; women with acetowhite lesions in the transformation zone can be treated with cryotherapy, in the same visit if treatment is available on‐site. Screening with Pap testing is not recommended unless an existing cytology program meets quality indicators for training, high coverage, and follow‐up 19.

HPV testing has achieved demonstrable reductions in cervical cancer incidence and mortality. A large randomized trial in India found that a single round of HPV testing in women over age 30 years reduced advanced cervical cancer incidence and mortality by 50%, whereas VIA and Pap testing did not yield significant reductions in disease burden 5. Other studies in India have found VIA to yield modest reductions; Sankaranarayanan and colleagues found that one round reduced incidence by 25% and mortality by 35%^20^, whereas Shastri and colleagues found that 4 rounds reduced only mortality by ~30% 21, likely due to detecting cancer at earlier stages rather than prevention of invasive cancer. A randomized trial evaluating HPV versus VIA screen‐and‐treat approaches found that the HPV screen‐and‐treat approach led to greater reductions in the prevalence and incidence of cervical intraepithelial neoplasia grade 2 and higher (CIN2+) over 36 months of follow‐up 6.

The demonstrated health impact of HPV testing is the result of high sensitivity to detect precancer and infections destined to become precancer, which is critical when the number of lifetime screening opportunities is low and the consequences of a false negative are potentially serious. HPV specimens can be collected by a provider (provider‐collection of cervical samples) or by the woman herself (self‐collection of vaginal samples). Studies of test performance have validated the *care*HPV test—a low‐cost HPV test with basic laboratory requirements—in low‐resource settings for both provider‐ and self‐collection 22, 23, with provider‐collection demonstrating slightly higher sensitivity to detect precancer.

Despite the high sensitivity and resulting effectiveness of an HPV screen‐and‐treat approach under study conditions, HPV test kits and laboratory processing systems are costly 24. Conventional HPV testing (including *care*HPV) requires at least two visits—first, for administration of the screening test, and second, for delivery of results and treatment of HPV‐positive women. High loss to follow‐up rates will undermine program success if many women do not receive necessary treatment. Realistically, even VIA may require multiple visits, as cryotherapy is unlikely to be available at all primary health facilities. Optimization of screening effectiveness will need to consider these tradeoffs of costs, number of visits, and achievable compliance with recommended follow‐up, as well as test sensitivity and specificity for each screening test or delivery mechanism in a given setting.

Mathematical simulation models of disease can integrate biologic, epidemiologic, economic, and behavioral data to project the long‐term health and economic impact of interventions 25. Increasingly, these models are used to inform decision making by quantifying potential tradeoffs between resource utilization, feasibility, and effectiveness. We performed a series of comparative‐ and cost‐effectiveness analyses using cost and test performance data from the PATH demonstration projects in India (Hyderabad), Nicaragua (Masaya Province), and Uganda (Kampala) to inform country‐specific mathematical simulation models of HPV infection and cervical cancer. Each model was calibrated to country‐specific epidemiologic data and used to project health and economic outcomes associated with various screening tests (HPV testing, VIA, and Pap testing), algorithms (screening age, screening interval, number of screens per lifetime, triage testing for screen‐positive women), and delivery strategies (number of visits per screening episode, HPV self‐ vs. provider‐collection). We summarize qualitative insights here.

Our model results indicate that the most critical ages for screening fall within WHO guidelines across settings with varying burdens of HPV and cervical cancer 16. Screening once or three times in a woman's lifetime with HPV testing (provider‐collection) at 70% coverage with linkage to treatment may reduce cervical cancer risk by ~25–50%. Regardless of the number of lifetime screens, cancer risk reductions were 3–5% lower for HPV self‐collection at the clinic, due to slightly reduced test sensitivity. Despite our optimistic modeling assumption that VIA screen‐and‐treat could usually be delivered in a single visit, whereas HPV testing required at least two visits, VIA yielded lower reductions in cancer risk due to reduced test sensitivity, which impacts screening effectiveness more than test specificity when screening opportunities are limited. Pap testing was the least effective strategy, due to both low sensitivity and the higher number of required visits. When all screening tests were compared in each country, only HPV testing was considered very cost‐effective, with incremental cost‐effectiveness ratios for screening three times in a lifetime below per capita GDP (a common benchmark for cost‐effectiveness)^26^ in each setting.

## Achieving High Screening Coverage

The WHO recommends that screening begin at 30 years of age, with priority given to maximizing population screening coverage rather than maximizing the number of screening tests in an individual woman's lifetime 19, 27. Details regarding the target ages and interval between screenings (within the window of screening eligibility) are left to country‐level decision makers, who may be guided by local disease burden, costs, and infrastructure.

An analysis of population‐based surveys from 57 countries found that the average cervical cancer screening coverage in developing countries is 19% among women aged 25–64 years, compared to 63% in developed countries 15. Screening in LMICs tends to occur opportunistically during contact with the health system, rather than through national screening programs that target women of a particular age 28. Yet even opportunistic screening occurs rarely in LMICs, due to the logistical barriers (including costs, transportation, and time away from work and family obligations) that prevent women from accessing the clinic, as well as lack of supplies and trained providers to conduct screening. Barriers to screening exist even for VIA, which requires minimal supplies and no laboratory. A WHO‐supported VIA demonstration project in six countries, which targeted all women between aged 30 and 50 years within designated catchment areas, achieved very low coverage over the 3 years of the study 29. Furthermore, VIA requires intensive provider training and experience to achieve acceptable test sensitivity, raising obstacles to scalability.

A particular advantage of HPV testing is that self‐collection of HPV specimens circumvents the need for a pelvic examination, potentially increasing acceptability and easing the burden on health systems by shifting the task of screening to women and community health workers. In Argentina, when community health workers visited women at home and offered the opportunity to self‐collect an HPV sample, screening uptake was 86%, compared to only 20% among women who were advised to attend a health clinic for screening 30. A study in rural areas of Mexico with limited access to health facilities found nearly perfect participation among women offered the opportunity to self‐collect at home, up 11% relative to those invited to the clinic for Pap testing 31. In low‐ and lower‐middle‐income settings, screening uptake increased from 54% to 72% when women in India were offered home‐based self‐collection rather than screening at the clinic 32, whereas a randomized trial in Uganda found that 99% of women approached for self‐collection at home or work participated (compared to 48% invited to the clinic for screening with VIA) 33.

With respect to screening coverage, well‐intentioned screening guidelines in many countries prescribe screening at routine intervals or a certain number of times per lifetime without acknowledging the reality that few women have access to any screening opportunity. When we used the Uganda model to examine the tradeoff between expanding coverage for a single lifetime screen versus increasing screening frequency to two or three times in a lifetime for a select group of women, we found that—when existing screening coverage was low (i.e., 30%)—expanding access to screening may lead to greater population‐level health gains, greater reduction in health disparities, and could be very cost‐effective, with an incremental cost‐effectiveness ratio below per capita GDP 17. At higher baseline coverage levels (i.e., 50–70%), screening three times in a lifetime is likely to yield greater reductions in cancer risk than screening once in a lifetime at higher coverage levels, and both strategies are projected to be very cost‐effective. These findings offer insight for screening program architects and implementers in low‐resource settings with a high burden of cervical cancer—namely, that if present screening coverage is low, concentrating limited resources on expanding access to once in a lifetime screening may yield greater health benefits and greater equity for public health dollars than screening a smaller proportion of women multiple times in a lifetime.

We also used the Uganda model to explore the efficiency and effectiveness of a onetime, community‐based HPV self‐collection campaign under different uptake scenarios. We found that, if most HPV‐positive women can be successfully navigated to treatment, HPV self‐collection is a very cost‐effective alternative to provider‐collection at the clinic, despite slightly reduced test sensitivity 18. When community‐based self‐collection was associated with screening coverage gains of 15–20%, it was more effective than provider‐collection. This finding offers assurance that the slight decrement in test performance associated with self‐collection may be offset if screening uptake increases sufficiently. Furthermore, these modeling results offer supporting evidence that community‐based HPV self‐collection leading to increased screening uptake in hard‐to‐reach populations may be cost‐effective. Implementation studies will be needed to determine setting‐specific acceptability, feasibility, and costs in particular hard‐to‐reach populations.

## Managing Screen‐Positive Women

The management of screen‐positive women poses challenges for screening programs in LMICs. According to WHO guidelines, treatment with either cryotherapy and/or loop electrosurgical excision procedure (LEEP) are essential components of screen‐and‐treat programs 19, 34, yet cryotherapy equipment and gas are costly and in short supply. As a result, women may need to be referred to higher level facilities, reducing compliance with recommended follow‐up. In a WHO VIA demonstration project in 6 African countries, only 60.9% of women eligible for cryotherapy received it; of those who received cryotherapy, only 39.1% received it on the same day as screening 29. Several studies with higher same‐day treatment rates following VIA were conducted in urban settings or at secondary and tertiary facilities 35, 36, and findings may not be generalizable to basic primary health facilities when included as part of a national screening program.

With HPV testing, the high prevalence of oncogenic HPV infections coupled with the relatively low prevalence of precancer leads to a high “false positive rate,” as few women who test positive for HPV will have or go on to develop precancer or invasive cancer in the future. An HPV‐based screening program that sends all HPV‐positive women to cryotherapy may lead to overtreatment. While there are no data to suggest that overtreatment with cryotherapy is harmful to women 37—in fact, it may slightly reduce the risk of future HPV infection 6—the procedure is costly and inflicts a burden on the health system. For HPV‐positive women, triage testing with VIA has been recommended to reduce the number of women referred to treatment 19. Limited data on the performance of VIA triage in HPV‐positive women suggest substantial degradation and uncertainty in performance, detecting only 25–67% of CIN2+ 38, 39, 40, 41.

Linking screen‐positive women to treatment is essential if the health benefits of screening are to be realized. While the primary advantages of VIA are its low cost and logistical advantages that theoretically facilitate a single‐visit approach, the evidence from demonstration projects and pilot studies indicates that its use as a single‐visit strategy may be limited to urban areas and higher level facilities 29, 35, 36. HPV testing, on the other hand, is not only associated with a higher cost screening test, but also with the higher costs of referring more women to treatment, burdening health systems that are already stressed. While VIA triage of HPV‐positive women may reduce this burden, it will also likely reduce the health benefits of screening, and may actually lead to greater economic costs in the long run through necessary follow‐up of HPV‐positive/VIA‐negative women.

We used the India, Nicaragua, and Uganda models to estimate the comparative‐ and cost‐effectiveness of HPV testing with VIA triage versus HPV testing alone and found that, even under the most optimistic assumptions about VIA performance, HPV testing alone was both more effective and more cost‐effective in Nicaragua and Uganda. In India, HPV testing with VIA triage had the potential to be a cost‐effective alternative to HPV testing alone when we assumed VIA triage test sensitivity was higher than suggested by much of the literature, but yielded lower reductions in cancer risk and was thus a less effective strategy. These findings reinforce the need for more accessible treatment options that can be readily delivered in all primary health facilities or mobile clinics in LMICs, averting the need for triage testing. Alternatively, low‐cost triage strategies with better ability to predict risk of precancer and cancer are needed.

## Opportunities for Efficient and Equitable Programming: Summarizing Insight from Mathematical Simulation Models

Each of the modeling analyses we summarize here is associated with its own limitations (documented elsewhere) 16, 17, 18, which may include sparse data on programmatic costs and costs associated with scale‐up; misspecification of screening algorithms that have not yet been implemented in a given setting; and limitations in the model calibration approach. Despite limitations, modeling results add to the evidence base that once in a lifetime screening with HPV testing can yield substantial reductions in cervical cancer incidence and mortality. Self‐collection of HPV samples may dramatically increase screening uptake while still achieving high sensitivity for precancer. A remaining challenge will be navigating HPV‐positive women to treatment, given the lack of effective, low‐cost triage tests available; however, new treatments such as thermocoagulation—currently undergoing validation in the field—are smaller, portable, and do not require gas 42, 43, 44. Innovative delivery mechanisms will be needed to ensure that these technologies reach women in need in a cost‐effective manner.

In addition to providing estimates of the health and economic impact of screening strategies, we used the models to determine the economic costs that could be additionally incurred for a new point‐of‐care HPV test to be cost‐effective; this is known as the incremental net monetary benefit (INMB). We found that, if screen‐positive women could subsequently be navigated to treatment, the economic value of an HPV test with same‐day results was high, particularly in settings with low rates of compliance to recommended follow‐up. Thus, in addition to providing qualitative insights across countries with diverse epidemiologic profiles and quantitative insights within a particular country, the models can estimate the value of improvements in screening practice 45, whether new technologies or interventions to improve compliance.

Through expanding access to HPV screen‐and‐treat programs that invoke innovative service delivery mechanisms, we have the tools to substantially reduce the global burden of cervical cancer. Lessons from small‐scale demonstration projects should be applied to implementation, with programs continually evaluated and adapted as roll‐out occurs. Modeling analyses can continue to evaluate the cost‐effectiveness of particular strategies, and will be further enriched as data from real‐world implementation become available. Strengthening primary health systems in low‐resource settings is difficult work, but imperative if we are to address health disparities and the growing burden of noncommunicable diseases in LMICs 46. With political will and coordinated efforts among governments, international organizations, and donors, and the prompt commitment of resources and technical support, cervical cancer can become a public health success story.

## Conflicts of Interest

NGC, VT, MM, and JJK declare no conflicts of interest. JJ was the director of the START‐UP demonstration projects and received all tests used in the study as a donation from QIAGEN. JJ was the co‐owner and Deputy Manager of Onco Prev International, a Peruvian company, from 2012 through March 2017. Onco Prev International offers cervical cancer screening services and in 2016 also began positioning for distribution of medical devices including colposcopes and the Liger thermocoagulator. Onco Prev International did not commercialize any medical instrument during the time Dr. Jeronimo was part of the company.
